# Integrated Bioinformatics Analysis Identifies NCAPG2 as an Immune-Related Prognostic Biomarker in Breast Cancer

**DOI:** 10.14740/wjon2768

**Published:** 2026-06-25

**Authors:** Jia Li, Qian Wang, Hao Qiao

**Affiliations:** a Health Management Department, The Second Affiliated Hospital of Xi’an Jiaotong University, Xi’an 710004, China; b Department of Thyroid Surgery, The Second Affiliated Hospital of Xi’an Jiaotong University, Xi’an 710004, China

**Keywords:** Bioinformatics analysis, Prognosis predicting, Breast cancer, Non-SMC condensin II complex subunit G2, Immune infiltration

## Abstract

**Background:**

Non-SMC condensin II complex subunit G2 (NCAPG2) is an important molecule in regulating chromosome segregation of mitosis and acts as an oncogene and biomarker in various tumors. This study aimed to explore the role of NCAPG2 in breast cancer (BC).

**Methods:**

The mRNA and protein expression of NCAPG2 was explored in the Gene Expression Profiling Interactive Analysis (GEPIA), Tumor Immune Estimation Resource (TIMER), Human Protein Atlas (HPA), and bc-GenExMiner databases. Survival analyses were performed using the Kaplan–Meier plotter and bc-GenExMiner databases. We used the COSMIC, CistromeDB, and cBioPortal databases to analyze the transcriptional regulation and genetic alteration. Function enrichment analyses were performed with CancerSEA and Metascape database.

**Results:**

NCAPG2 expression was significantly higher in BC than normal samples. NCAPG2 expression was positively correlated with the Scarff–Bloom–Richardson (SBR) grade, HER2 status, lymph nodal status, TP53 and BRCA1/2 mutation, and Nottingham prognostic index (NPI), while negatively correlated with age, ER status, and PR status. Survival analyses indicated that overexpressed NCAPG2 was associated with the adverse prognosis of BC. Gene Set Enrichment Analysis (GSEA) and function enrichment analyses of single-cell and co-expressed genes of NCAPG2 consistently showed that NCAPG2 was involved in cell cycle, immune, DNA damage, cancer-related pathways, proliferation, and DNA repair. The result of TIMER showed that NCAPG2 was associated with infiltrating immune cells and the exhausted T cell phenotype, such as CD8^+^ T cells, PD-1, CTLA4, and TIM-3.

**Conclusion:**

We found that the mRNA and protein expression of NCAPG2 was upregulated in BC. Overexpressed NCAPG2 might act as an adverse prognosis biomarker in BRCA and had a distinct function in the cell cycle, proliferation, and immune infiltration.

## Introduction

Female breast cancer (BC) ranks first in diagnosed and fifth in cancer-related deaths worldwide for 36 cancers in 185 countries [1]. With diagnosis and treatment progress, BC prognosis has been greatly improved [2, 3]. However, due to the heterogeneity of tumor microenvironment (TME) or intrinsic and inherent features of tumor cells, drug resistance problems emerge one after another [4, 5]. Thus, it is very significant to explore new treatment targets and prognostic biomarkers for BC.

The non-SMC condensin II complex subunit G2 (NCAPG2) is a component of the condensin II complex, and plays a significant role in regulating correct chromosome segregation during mitosis [6]. In recent years, several studies have indicated that NCAPG2 was involved in various biological functions and the development of cancers, such as cell proliferation, survival, and metastasis [7, 8]. NCAPG2 could promote cell proliferation and migration in liver cancer by the STAT3 and NF-κB/miR-188-3p pathways [9, 10]. In lung adenocarcinoma, NCAPG2 could be regulated by miR-188-3p and regulate the cell cycle and proliferation [11, 12]. NCAPG2 could facilitate glioblastoma cells’ malignancy and xenograft tumor growth [13]. However, the expression level, prognostic value, and function of NCAPG2 remain unclear in BRCA.

In this study, we explored the expression level, diagnostic and prognostic values, and the potential function of NCAPG2 in BRCA for the first time by integrating various bioinformatics analyses.

## Materials and Methods

### Patients and datasets

We extracted the survival and mRNA expression data of invasive breast carcinoma (BRCA) patients from the GDC Data Portal [14]. We converted the mRNA data into TPM format for further analysis. We included patients diagnosed with primary invasive breast carcinoma (BRCA) who had complete mRNA expression data and survival information available from the TCGA database.

### Tumor Immune Estimation Resource (TIMER)

TIMER [15] is a powerful online interactive website to analyze tumor immune and gene expression [16]. We used it to analyze the expression of NCAPG2 in various cancer types and study the correlation between NCAPG2 expression with immune cells infiltrating levels and the potential transcription factors (TFs) in BRCA.

### Gene Expression Profiling Interactive Analysis (GEPIA)

GEPIA [17] is an online database tool including gene expression, correlation, and survival analyses [18]. We used it to explore the expression level of NCAPG2 in BRCA tissues and normal tissues based on TCGA and GTEx data.

### The Human Protein Atlas (HPA)

The HPA database [19] is a powerful online interactive website to analyze human protein profiles in cells and tissues [20, 21]. We used it to explore the protein expression of NCAPG2 in BRCA.

### Bc-GenExMiner

Bc-GenExMiner [22] is an online interactive tool for studying BRCA including the database from TCGA, METABRIC, and SCAN-B [23]. We used it to explore the expression levels of NCAPG2 in different sub-types and the prognosis value of NCAPG2 in breast cancer.

### Kaplan–Meier plotter

The Kaplan–Meier plotter database [24] is a powerful online interactive website for cancer prognosis analysis [25]. We used it to analyze the prognosis value of NCAPG2 in overall survival (OS), relapse-free survival (RFS), and distant metastasis-free survival (DMFS) of BRCA. We further analyzed the prognosis values of the TFs and 10 hub genes.

### Mutant and transcriptional regulation analysis of NCAPG2 in BRCA

The mutation frequency, mutation sites of NCAPG2 in BRCA, and the prognosis value of mutation were explored in the cBioportal database [26, 27]. Besides, the Catalogue of Somatic Mutations in Cancer (COSMIC) database [28] further revealed the mutation and substitutional types of NCAPG2 [29]. We further predicted the potential TF of NCAPG2 in BC with the “Toolkit” modular of the CistromeDB database [30, 31].

### Single-cell functional analysis and Gene Set Enrichment Analysis (GSEA)

CancerSEA [32] is a database depicting 14 functional states of 41,900 cancer cells from 25 tumor types at the single-cell level [33]. We used it to query the functional status of NCAPG2 in BRCA in this study, such as stemness, DNA repair, and proliferation. GSEA is a desktop application for enrichment analysis to reveal the differences of *a priori* defined genes set between two biological states [34]. We used it to analyze the functional differences between the high-expression and low-expression NCAPG2 groups. We downloaded the annotated gene set “c2.cp.kegg.v7.4.symbols.gmt” from the Molecular Signatures Database. The significance thresholds were set at false discovery rate (FDR) < 0.25 and P < 0.05.

### Prediction of co-expressed genes of NCAPG2, construction of protein-protein interactions (PPI) network, and identification of hub genes

The positive co-expression genes of NCAPG2 were identified in the cBioPortal database [26, 27]. We used the Metascape database [35], Gene Ontology (GO), and Kyoto Encyclopedia of Genes and Genomes (KEGG) to explore the biological functions of the co-expression genes. We constructed the PPI network in the STRING database [36] and identified 10 hub genes with the Cytoscape (version 3.9.0).

### Statistical analyses

We applied R software (version 4.0.5 [37]) and the online bioinformatics databases for all statistical analyses and regarded two-tailed P < 0.05 as statistically significant. The R package “pROC” was used to plot the ROC plot of NCAPG2 in BC. The R package “clusterProfiler” was conducted to perform GO and KEGG analyses of the co-expressed genes. The significance level in all figures was set as *P ≤ 0.05, **P ≤ 0.01, ***P ≤ 0.001, and ns = P > 0.05.

## Results

### The mRNA and protein expression of NCAPG2 in BC

**Figure 1:**
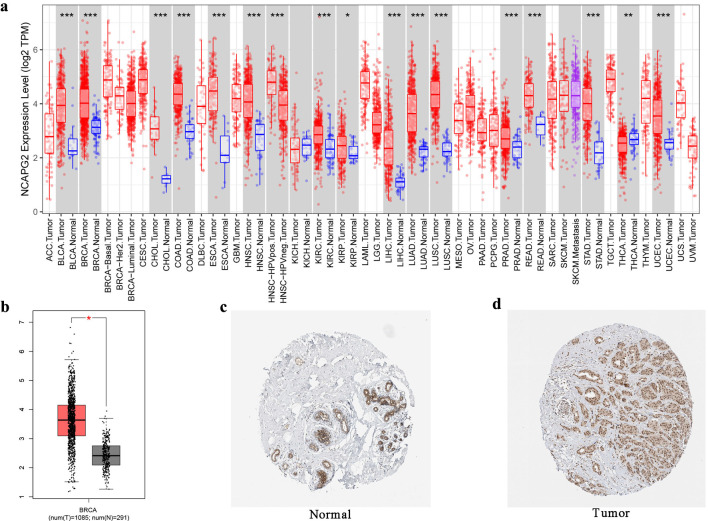
The mRNA and protein expression of NCAPG2 in BRCA. (a) NCAPG2 was upregulated in 18 cancer types in the TIMER database. (b) The mRNA expression of NCAPG2 in BRCA in the GEPIA database. (c, d) The protein expression of NCAPG2 in normal tissue and breast cancer tissue in the HPA database.

We explored the mRNA and protein expression of NCAPG2 in the three online databases. Figure 1a shows that NCAPG2 expressed much higher in BRCA and other 17 kinds of tumors than normal samples. Consistently, the results of the GEPIA database further certified the significantly higher mRNA expression of NCAPG2 in BRCA tissues (Fig. 1b). The HPA database showed that the NCAPG2 protein was the medium expression in the normal tissue, while high expression in BRCA tissue (Fig. 1c, d). The above results showed that the expression of NCAPG2 was upregulated in BC tissues.

### Association between NCAPG2 expression and the clinical characteristics

**Figure 2:**
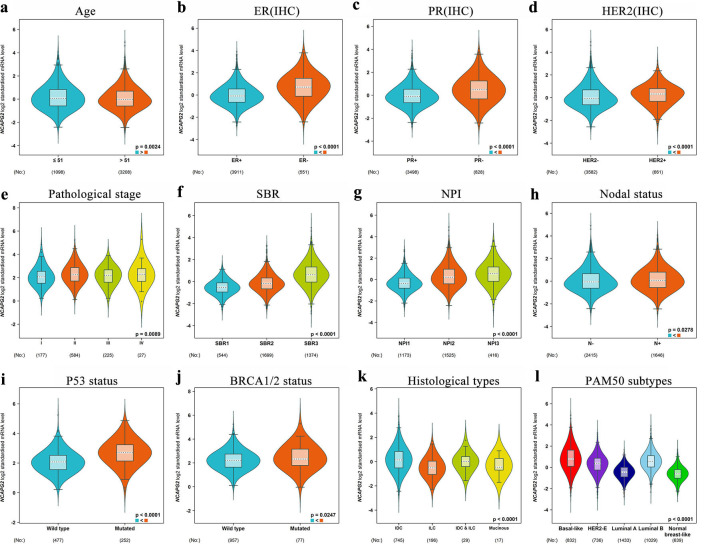
The transcription level of NCAPG2 in the subgroup of BRCA patients. The boxplots of BRCA patients classified by age (a), ER (b), PR (c), HER2 (d), pathological stage (e), SBR (f), NPI (g), node status (h), TP53 mutation status (i), BRCA1/2 mutation status (j), histological type (k), and PAM50 subtypes (l).

We further explored the NCAPG2 expression in BRCA patients with different clinical characteristics using the bc-GenExMiner database. NCAPG2 expressed higher in ≤ 51-year group compared with > 51-year group (Fig. 2a). Patients with positive HER-2 status and negative ER and PR status had a higher expression level of NCAPG2 (Fig. 2b–d). Patients at pathological stage II had a higher level than pathological stage I and stage III, but no significant differences were observed among other stages (Fig. 2e). With the increase of SBR grade and NPI, the expression of NCAPG2 was significantly upregulated (Fig. 2f, g). Patients with positive nodal status (N), TP53 mutation, and BRCA1/2 mutation had an increased level of NCAPG2 (Fig. 2h–j). As to the histological types, invasive ductal carcinoma had a higher expression level than invasive lobular carcinoma and mucinous (Fig. 2k). Besides, the basal-like subtype had the highest level, followed by luminal B, HER2-E, and luminal A subtypes (Fig. 2l). The above results indicated that increased expression of NCAPG2 was related to worse clinical grade and subtype.

### Prognostic and diagnostic roles of NCAPG2 in BRCA

**Figure 3:**
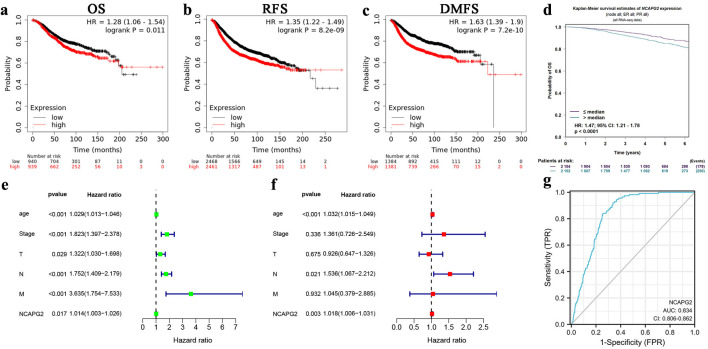
Prognostic and diagnostic roles of NCAPG2 in BRCA. Survival curve of differential NCAPG2 expression in BRCA in the Kaplan–Meier plotter database (a–c) and the bc-GenExMiner (d). Univariate (e) and multivariate (f) Cox analyses of clinicopathological parameters and NCAPG2 in BRCA. (g) The ROC curve of NCAPG2 in BRCA.

We analyzed the prognostic and diagnostic roles of NCAPG2 in the online bioinformatics database and R software. The result of Kaplan–Meier plotter database showed that increased NCAPG2 expression was related to worse OS (hazard ratio (HR) = 1.28 (1.06–1.54), P = 0.011), RFS (HR = 1.35 (1.22–1.49), P = 8.2 × 10^−9^), and DMFS (HR = 1.63 (1.39–1.9), P = 7.2 × 10^−10^) (Fig. 3a–c). Consistently, we observed the similar results in the bc-GenExMiner (OS: HR = 1.47 (1.21–1.78), P < 0.0001) (Fig. 3d). The univariate and multivariate analyses indicated that NCAPG2 was the independent prognosis factor for BRCA (univariate: HR = 1.014 (1.003–1.026), P = 0.017; multivariate: HR= 1.018 (1.006–1.031), P = 0.003) (Fig. 3e, f). The area under the ROC curve (AUC) was 0.834, indicating the capacity of NCAPG2 for distinguishing BRCA tissues from normal tissues (Fig. 3g). These results showed the prognostic and diagnostic capacities of NCAPG2 in BRCA.

### A nomogram based on NCAPG2 to predict the survival probability of BRCA

**Figure 4:**
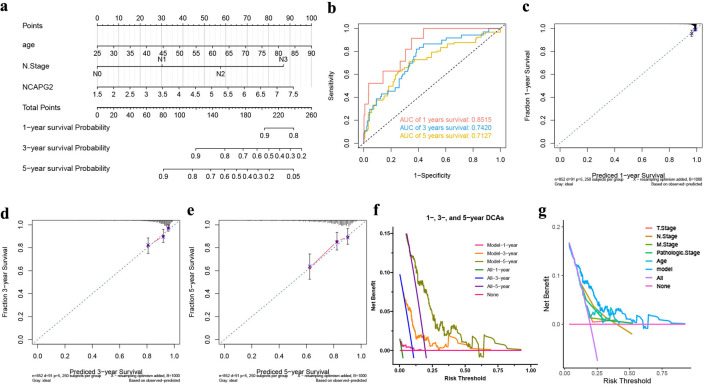
A nomogram based on NCAPG2 to predict the survival probability of BRCA. (a) A nomogram for predicting BRCA prognosis with NCAPG2 expression. The 1-, 3-, and 5-year ROC curves (b), calibration curves (c–e), and DCA curves (f). (g) The DCA curves of the nomogram and different clinicopathological parameters.

To predict the 1-, 3-, and 5-year survival probability of BRCA, we constructed a nomogram by combining all the independent prognosis factors, including the expression of NCAPG2, age, and N stage (Fig. 4a). The AUCs of 1-, 3-, and 5-year ROC curves were 0.8515, 0.7420, and 0.7127 (Fig. 4b). The 1-, 3-, and 5-year calibrate curves and decision curve analysis (DCA) curves of the nomogram indicated its accuracy and benefit (Fig. 4c–f). Figure 4g shows that our model could provide more benefits than other clinicopathological parameters. All these results indicated the importance of NCAPG2 in BRCA prognosis prediction.

### Mutant and transcriptional regulation analysis of NCAPG2 in BRCA

**Figure 5:**
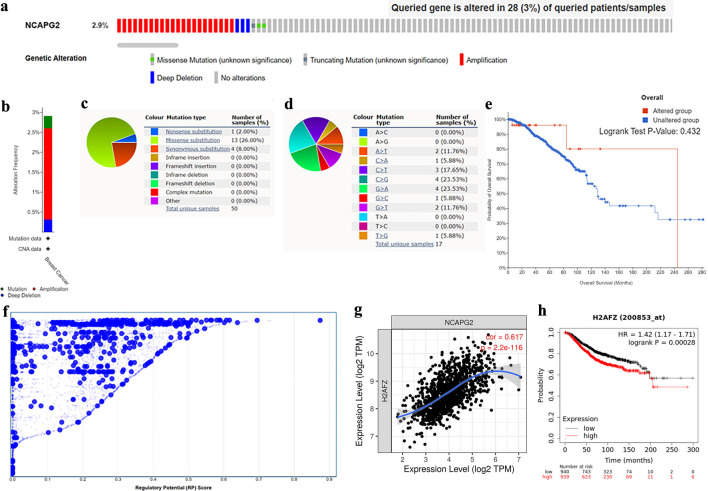
Mutant and transcriptional regulation analysis of NCAPG2 in BRCA. (a, b) The overview of alteration types and corresponding frequencies of NCAPG2. (c, d) The summary of mutation types and substitutional types of NCAPG2 in the COSMIC database. (e) The Kaplan–Meier survival curve in different mutation status. (f) The potential transcription factors of NCAPG2 in breast cancer (10 k distance to TSS). (g) The correlation of H2AFZ and NCAPG2 expression in BRCA in the TIMER database. (h) The prognosis value of H2AFZ in BRCA in the Kaplan–Meier plotter database.

Both genetic mutation and transcriptional regulation could influence gene expression. We explored the mutation of NCAPG2 in BRCA based on the cBioPortal and COSMIC databases. Figure 5a, b shows that the frequency of NCAPG2 mutation was 2.9% in 986 BRCA patients (TCGA, Firehose Legacy), and amplification was the main mutation type. We further explored the mutation types of NCAPG2 with the COSMIC database. Figure 5c shows that missense substitution occurred in 26.00% of the 50 samples. Figure 5d shows that mutations mainly occurred in G>A and C>G, followed by C>A, G>T, and A>T. Figure 5e shows no correlation between the altered status of NCAPG2 and the prognosis of BRCA (P = 0.432).

Furthermore, to find the molecules that might regulate the expression of NCAPG2, we predicted the TFs of NCAPG2 in the breast with the CistromeDB database. We found that NONO, CTCF, BRD4, SP1, and H2AFZ were the top five TFs that possessed the regulatory potential (Fig. 5f). Further co-expression and analyses found that H2AFZ was the most potential TF. It was positively correlated with NCAPG2 expression and related to the adverse prognosis of BRCA (Fig. 5g, h), while the other four TFs had inconsistent correlations (Supplementary Material 1, wjon.elmerpub.com).

### Single-cell functional analysis and GSEA

**Figure 6:**
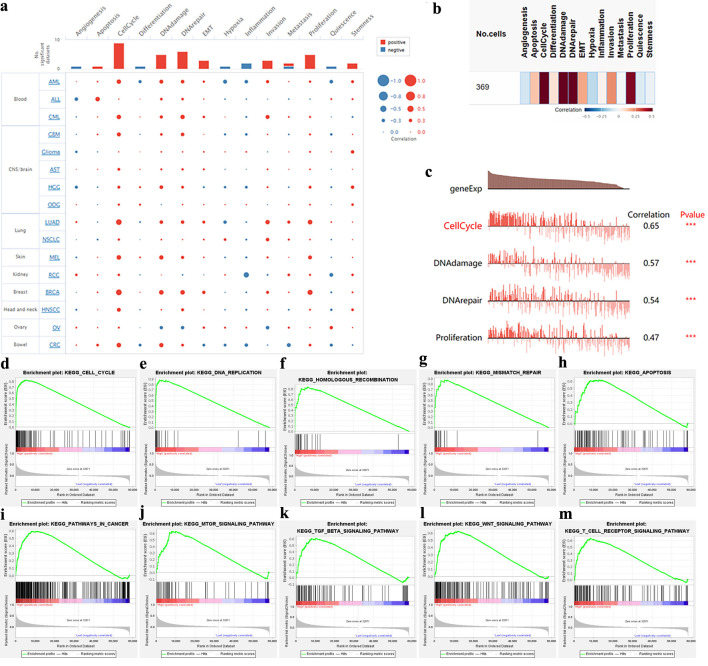
Single-cell functional analysis of NCAPG2. (a, b) The NCAPG2-related functional states in various cancers and BRCA. (c) The four functional states significantly correlated with NCAPG2. (d–m) Parts of the results of GSEA.

We explored the role of NCAPG2 in BRCA with GSEA and single BRCA cell functional analysis. The results showed that NCAPG2 was mainly involved in the cell cycle, DNA damage, epithelial–mesenchymal transition (EMT), invasion, proliferation, and DNA repair in various cancers (correlation > 0.2) (Fig. 6a). In particular, the correlations between BC and multiple functions were the highest among various cancers, including cell cycle, DNA damage, DNA repair, and proliferation (Spearman’s coefficients = 0.65, 0.57, 0.54, and 0.47 respectively; P < 0.001) (Fig. 6b, c). The result of GSEA showed that there were 121 pathways enriched in the high-expression NCAPG2 group, which were mainly associated with cancer, cell cycle, DNA repair, and immune such as “KEGG_WNT_SIGNALING_PATHWAY”, “KEGG_DNA_REPLICATION”, “KEGG_TGF_BETA_SIGNALING_PATHWAY”, “KEGG_CELL_CYCLE”, “KEGG_MTOR_SIGNALING_PATHWAY”, “KEGG_PATHWAYS_IN_CANCER”, “KEGG_MISMATCH_REPAIR”, “KEGG_T_CELL_RECEPTOR_SIGNALING_PATHWAY”, “KEGG_APOPTOSIS”, and “KEGG_HOMOLOGOUS_RECOMBINATION” (Fig. 6d–m, Supplementary Material 2, wjon.elmerpub.com). But there were only four pathways enriched in the low-expression NCAPG2 group (Supplementary Material 3, wjon.elmerpub.com). All these results indicated the potential carcinogenic effect of NCAPG2 in BC.

### Prediction of co-expressed genes of NCAPG2, construction of PPI network, and identification of hub genes

**Figure 7:**
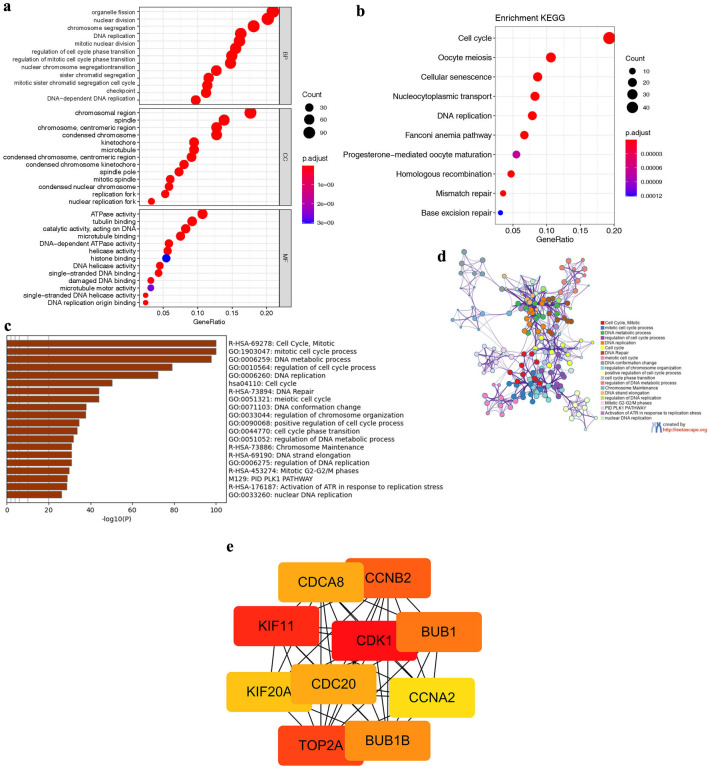
The co-expressed genes of NCAPG2, PPI network, and hub genes. (a) The result of GO analysis of the co-expressed genes, including biological progress, cellular component, and cellular component. (b) The result of KEGG analysis of the co-expressed genes. (c, d) The functional enrichment analysis results of the co-expressed genes in the Metascape database. (e) The 10 hub genes of the PPI network identified with Cytoscape.

We acquired 576 positive co-expression genes of NCAPG2 in BRCA from the cBioPortal database (Supplementary Material 4, wjon.elmerpub.com). GO analysis indicated that these genes participated in many biological processes, such as nuclear division, organelle fission, and cell cycle checkpoint (Fig. 7a). They played an essential part in the tubulin binding, helicase activity, and ATPase activity and acted as structural constituents in the chromosomal region, spindle, and kinetochore (Fig. 7a). KEGG analysis showed enrichment function in cell cycle, oocyte meiosis, cellular senescence, nucleocytoplasmic transport, and DNA replication (Fig. 7b). Furthermore, the results of the Metascape database also indicated the relationship among cell cycle, mitotic cell cycle progress, DNA metabolic, and DNA replication processes (Fig. 7c), and Figure 7d shows the clustering relationship among them. We further constructed the PPI network of the 576 co-expression genes and explored the 10 hub genes in Cytoscape, including KIF11, CDK1, TOP2A, CCNB2, BUB1B (SSK1), BUB1, CDC20, CDCA8, KIF20A, and CCNA2 (Fig. 7e). Supplementary Material 5 (wjon.elmerpub.com) shows that the 10 genes were all associated with the adverse outcome of BRCA.

### Association between NCAPG2 and immune infiltration in BRCA

**Figure 8:**
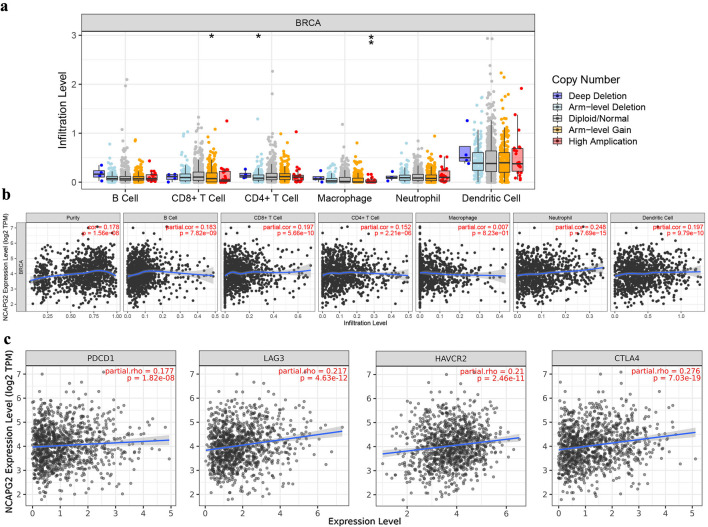
Association between NCAPG2 and immune infiltration in BRCA. (a) The correlation of the copy number of NCAPG2 and the infiltration levels of six immune cells. (b) The correlation between NCAPG2 expression and the infiltration levels of the immune cells. (c) The association of NCAPG2 expression and PD-1 (PDCD1), LAG3, TIM-3 (HAVCR2), and CTLA4.

The results of GSEA indicated that NCAPG2 was associated with immune-related pathways. We further analyzed the correlation in the TIMER database. Figure 8a shows that NCAPG2 copy number was associated with the infiltrating levels of CD8^+^ T cells, CD4^+^ T cells, and macrophage. Figure 8b shows that the NCAPG2 expression was positively correlated with B cells (cor = 0.183, P = 7.82 × 10^−9^), CD8^+^ T cells (cor = 0.197, P = 5.66 × 10^−10^), CD4^+^ T cells (cor = 0.152, P = 2.21 × 10^−6^), neutrophil (cor = 0.248, P = 7.69 × 10^−15^), and dendritic cell (cor = 0.197, P = 9.79 × 10^−10^). Additionally, NCAPG2 expression was positively correlated with the molecular markers of many immunological cells in BRCA (Table 1).

**Table 1:** The Correlation Between NCAPG2 Expression and the Molecular Markers of Immunological Cells

Description	Gene markers	BRCA (n = 1,093)			
		None		Purity	
		cor	P	cor	P
CD8^+^ T cell	CD8A	0.055	0.0663	0.188	***
	CD8B	0.019	0.532	0.139	***
T cell (general)	CD3D	−0.005	0.861	0.119	***
	CD3E	0.013	0.66	0.147	***
	CD2	0.067	0.0273	0.201	***
B cell	CD19	0.015	0.614	0.105	***
	CD79A	−0.009	0.775	0.092	**
Monocyte	CD86	0.137	***	0.226	***
	CD115 (CSF1R)	−0.025	0.413	0.059	0.0609
TAM	CCL2	0.093	**	0.183	***
	CD68	0.119	***	0.193	***
	IL10	0.202	***	0.287	***
M1 macrophage	INOS (NOS2)	0.16	***	0.118	***
	IRF5	0.058	0.0543	0.209	***
	COX2 (PTGS2)	0.14	***	0.144	*
M2 macrophage	CD163	0.238	***	0.323	***
	VSIG4	0.036	0.227	0.107	***
	MS4A4A	0.116	***	0.219	***
Neutrophils	CD66B (CEACAM8)	0.054	0.0738	0.046	0.151
	CD11B (ITGAM)	0.075	0.0133	0.138	***
	CCR7	0.048	0.11	0.17	***
Natural killer cell	KIR2DL1	0.056	0.062	0.111	***
	KIR2DL3	0.131	***	0.178	***
	KIR2DL4	0.158	***	0.224	***
	KIR3DL1	0.107	***	0.164	***
	KIR3DL2	0.089	**	0.174	***
	KIR3DL3	0.078	**	0.112	***
	KIR2DS4	0.08	**	0.14	***
Dendritic cell	HLA-DPB1	−0.167	***	-0.084	**
	HLA-DQB1	−0.066	0.0285	0.007	0.815
	HLA-DRA	0.015	0.619	0.122	***
	HLA-DPA1	−0.044	0.142	0.054	0.0917
	BDCA-1 (CD1C)	−0.132	***	−0.056	0.078
	BDCA-4 (NRP1)	0.027	0.375	0.104	***
	CD11c (ITGAX)	0.072	0.0166	0.16	***
Th1	T-bet (TBX21)	0.04	0.19	0.168	***
	STAT4	0.057	0.0589	0.181	***
	STAT1	0.343	***	0.181	***
	IFN-γ (IFNG)	0.138	***	0.389	***
	TNF-α (TNF)	0.215	***	0.231	***
Th2	GATA3	−0.08	*	0.252	***
	STAT6	0.017	0.584	0.003	0.917
	STAT5A	−0.046	*	−0.08	**
	IL13	0.07	*	0.051	0.0896
Tfh	BCL6	−0.03	0.317	−0.009	0.767
	IL21	0.177	***	0.228	***
Th17	STAT3	0.168	***	0.185	***
	IL17A	0.091	**	0.134	**
Treg	FOXP3	0.189	***	0.292	***
	CCR8	0.327	***	0.405	***
	STAT5B	0.01	0.733	0.039	0.215
	TGF-β (TGFB1)	−0.199	***	−0.127	***

Considering the potential oncogenic role of NCAPG2 in BRCA, we also accessed the relationship of NCAPG2 with exhausted T cell phenotype in the TIMER database. Figure 8c shows that the positive correlation between NCAPG2 and PD-1 (PDCD1) (cor = 0.177, P = 1.82 × 10^−8^), LAG3 (cor = 0.217, P = 4.63 × 10^−12^), TIM-3 (HAVCR2) (cor = 0.21, P = 2.46 × 10^−11^), and CLTA4 (cor = 0.276, P = 7.03 × 10^−19^). These results suggested that NCAPG2 might have a distinct function in immune infiltration and immune escape in BRCA.

### NCAPG2 expression and immune landscape in pan-cancers

**Figure 9:**
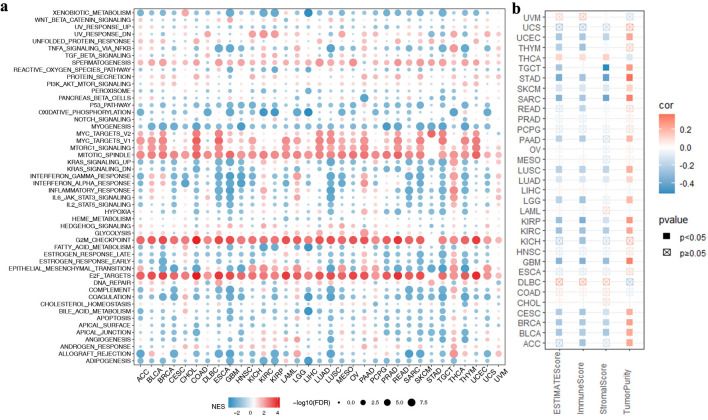
Associations between the NCAPG2 expression and immune landscape in pan-cancers. (a) The association between NCAPG2 expression and cancer-related pathways in pan-cancers. (b) The association between NCAPG2 expression and immune landscape in pan-cancers.

We intended to further clarify the correlation between NCAPG2 expression and immune infiltrating in pan-cancer. Firstly, the functional enrichment of high and low NCAPG2 expression in different cancers was investigated using GSEA. NCAPG2 expression was mainly positively connected with cancer-related activities such as MYC_TARGETS_V2, MYC_TARGETS_V1, MTORC1_SIGNALING, MITOTIC_SPINDLE, G2M_CHECKPOINT, and E2F_TARGETS, while was negatively connected with immune-related activities, such as INFLAMMATORY_RESPONSE, KRAS_SIGNALING_UP, and IL2_STAT5_SIGNALING (Fig. 9a). We further investigated the relationship between the NCAPG2 expression and the TME and found that NCAPG2 expression was negatively correlated with stromal, immune, and estimate scores in all 33 tumor types, while was positively correlated with tumor purity (Fig. 9b).

## Discussion

BC progression is correlated with the activation of dominant-acting oncogenes [38]. Although the mechanism of BC development has been revealed in the past, and novel chemotherapy, targeted therapy, and immunotherapy have improved its prognosis, it still ranks first (11.7%) in diagnosed cancer and fifth (6.9%) in cancer-related deaths worldwide in 2020 [1]. As an important molecule of mitosis, NCAPG2’s role in carcinogenicity in many cancers has been revealed in recent years. Here, we explored the expression level, diagnostic and prognostic values, and the function of NCAPG2 in BRCA with integrated various bioinformatics methods.

Our study first found that the mRNA and protein expression of NCAPG2 was higher in BRCA than in normal samples. NCAPG2 expression was higher in patients with ER and PR negative status, HER2 positive status, lymph node infiltration, TP53 mutation, BRCA1/2 mutation, and basal-like subtype. Furthermore, the NCAPG2 expression was positively correlated with SBR and NPI grades. Survival analyses showed that increased NCAPG2 expression had adverse prognoses, including OS, RFS, and DMFS. The ROC curve and the Cox analyses showed that NCAPG2 had an excellent diagnosis and independent prognosis value for BC. The nomogram based on NCAPG2 further indicated the important prognosis value of it. These results indicated that NCAPG2 was associated with an increased degree of malignancy in BC and might act as a diagnosis and prognosis biomarker of BRCA.

To explore the factors regulating the abnormal expression of NCAPG2, we analyzed the genetic mutant and transcriptional regulation of NCAPG2. There was a low mutant frequency of NCAPG2 (∼2.9%) in the cBioPortal database. We did not find the influence of mutation on BC survival. We further found that H2AFZ was the most potential TF. H2AFZ is one of the histone H2A variants and related to gene transcription regulation, chromosome segregation, and DNA repair [39, 40]. The previous study had shown that H2AFZ was upregulated in BRCA and related to lymph node metastasis and survival [41]. Overexpressed H2AFZ could promote tumor progression by regulating cell cycle, apoptosis, and EMT [42–44]. These results indicated that H2AFZ might regulate NCAPG2 expression in BRCA and then promote BC progression, but it needed to be further verified by experiments.

GSEA and the functional enrichment analysis of the single-cell and the positively co-expressed genes were carried out to unravel the biological functions of NCAPG2. The results showed that these genes were related to the cell cycle, DNA damage, EMT, invasion, proliferation, and DNA repair. The previous studies had demonstrated that NCAPG2 was involved in the cell cycle, invasion, proliferation, and DNA repair in other cancers [9, 11–13]. Furthermore, the result of GSEA showed many pathways related to cancer, cell cycle, DNA repair, and immune enriched in the high-expression NCAPG2 group (Supplementary Material 2, wjon.elmerpub.com). Thus, NCAPG2 might promote the development and progression of BRCA by Wnt, mTOR, P53, and TGF-β signaling pathways. These results further indicated the function of NCAPG2 that might act as an oncogene in BC.

As to the 10 hub genes of the PPI network, we found that they were all associated with the adverse prognosis of BRCA. The previous studies also had shown that the 10 genes were potential biomarkers in BC and other cancers. Top2A expression was a negative prognostic biomarker in BRCA, papillary thyroid cancer, and bladder cancer [45–47]. CDK1 and CCNA2 were cell cycle-associated proteins, and might be the potential therapeutic targets and prognostic biomarkers for BRCA, epithelial ovarian cancer, and pancreatic adenocarcinoma [48–50]. CDC20 was correlated with the adverse prognosis of TNBC and hepatocellular carcinoma, and inhibition of it could suppress the metastasis of TNBC [51, 52]. Previous studies also showed that CDCA8 and CCNB1 could act as promising molecular targets for BC [53, 54]. KIF11 could regulate the development and progression of BRCA [55]. KIF20A could promote BRCA, renal clear cell carcinoma, and medulloblastoma tumors [56–58]. Both BUB1 and BUB1B were spindle checkpoint genes, and they might be markers of BRCA with chromosomal instability [59]. The 10 hub co-expressed genes further confirmed the possibility of NCAPG2 as an oncogene for BRCA.

GSEA indicated that overexpressed NCAPG2 was associated with T cell, B cell, and natural killer cell signaling pathways. The development of a tumor involves the interaction between tumor and immune cells. Several studies have revealed that immune infiltrations had different prognosis values for patients with BRCA and could be detected and characterized in BC tissues [60]. The previous study had shown the association between high levels of tumor-infiltrating lymphocytes (TILs) and better prognosis and response to certain therapies [60]. But the presence of PD-1-positive TILs was related to the adverse prognosis in BRCA [61]. Studies have shown that LAG3 and TIM-3+ TILs could inhibit T cell activity [62, 63], while the combination of blocking immune checkpoints can obtain a better curative effect [64]. Our study found that NCAPG2 was related to the infiltrating levels of neutrophils, B cells, CD8^+^ T cells, CD4^+^ T cells, dendritic cells, and their molecular markers in BRCA. Furthermore, we also found a strong correlation between NCAPG2 and the exhausted T cell phenotype, including PD-1, CTLA4, LAG3, and TIM-3. NCAPG2 might lead to tumor immune escape by upregulating the immunosuppressive molecules. These results might indicate that targeting NCAPG2 could regulate immune escape and enhance the efficacy of BC immunotherapy.

However, our research still had a few limitations. The major limitation of this study is the lack of experimental validation using our own clinical samples or functional assays. All findings are derived from public databases and computational predictions. Therefore, the conclusions regarding NCAPG2 expression, prognosis, and immune association remain descriptive and hypothesis-generating. Independent validation in large clinical cohorts (qRT-PCR, immunohistochemistry) and mechanistic studies (cell lines, animal models) is essential to confirm the functional role of NCAPG2 in BC. Another limitation is that we did not perform subtype-stratified survival analysis due to the exploratory nature of this study; future studies should evaluate the prognostic value of NCAPG2 within each molecular subtype using larger, subtype stratified cohorts. Additionally, the present study is a single-cohort discovery analysis without a formal meta-analysis; future studies integrating multiple independent cohorts using methods such as pooled hazard ratios or summary ROC curves would further validate our finding.

### Conclusion

In conclusion, we found that the mRNA and protein expression of NCAPG2 was upregulated in BRCA. Overexpressed NCAPG2 might act as an adverse prognosis biomarker in BRCA and had a distinct function in cell cycle, proliferation, and immune infiltration.

## Supplementary Material

Suppl 1 The correlation of the other four potential TFs and NCAPG2 (A–D), and the prognostic value of them (E–H).

Suppl 2 The results of GSEA in the high expression NCAPG2 group.

Suppl 3 The results of GSEA in the low expression NCAPG2 group.

Suppl 4 The 576 positive co-expression genes of NCAPG2 in BRCA (cor > 0.4, P < 0.001).

Suppl 5 The prognosis analyses of the 10 hub genes (A–J).

## Data Availability

Data can be found at: https://portal.gdc.cancer.gov/repository. The data supporting the findings of this study are available from the corresponding author upon reasonable request.
