# Supplementary material for: Integrated Bioinformatics Analysis Identifies NCAPG2 as an Immune-Related Prognostic Biomarker in Breast Cancer
Source: World J Oncol. 2026 Jun 25;17(4):509–23. doi: 10.14740/wjon2768 (PMC13375428; doi:10.14740/wjon2768)

Suppl 1. The correlation of the other four potential TFs and NCAPG2 (A-D), and the prognostic value of them (E-H).


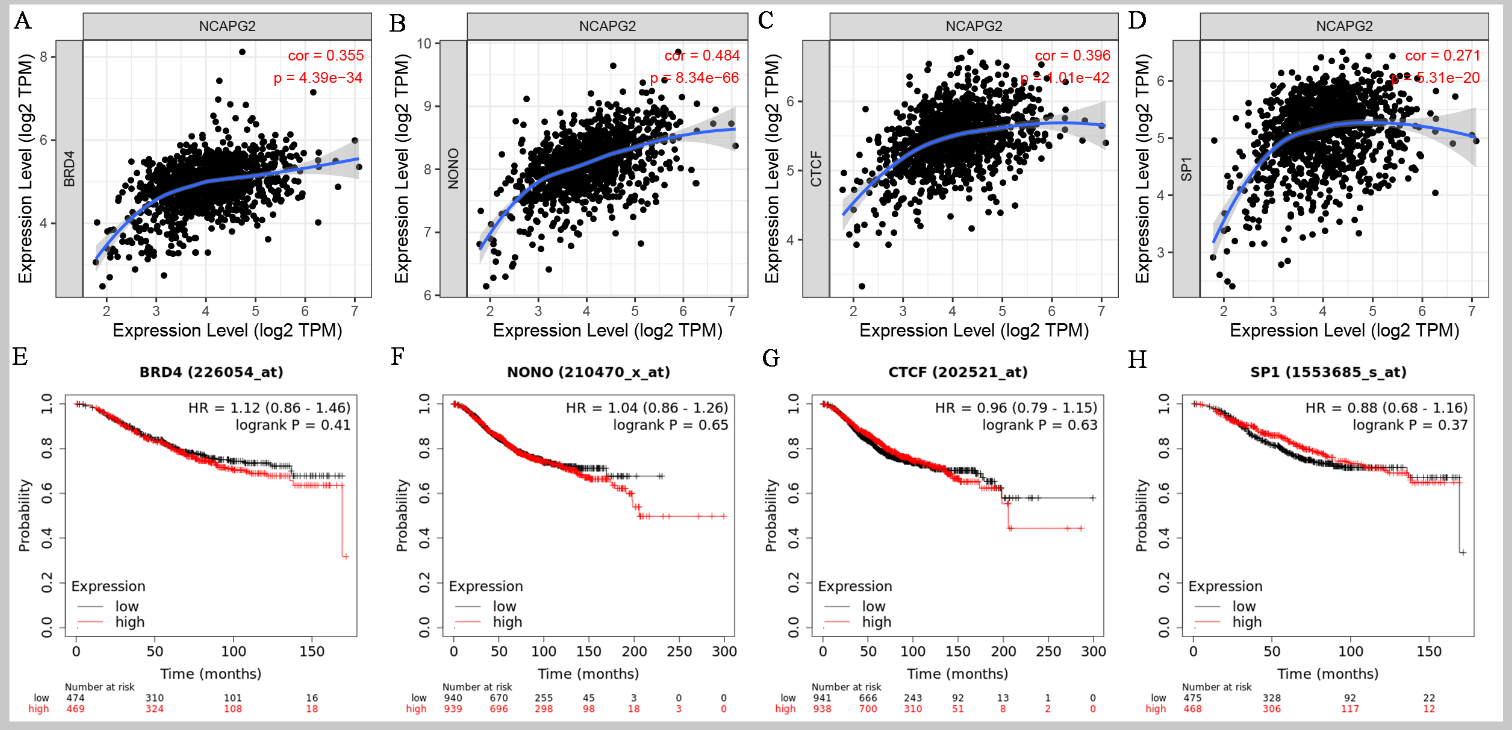

Supplement: Suppl 1 — The correlation of the other four potential TFs and NCAPG2 (A–D), and the prognostic value of them (E–H). [file wjon-17-04-509-s001.docx]
