# Supplementary figures and images for: Integrated Bioinformatics Analysis Identifies NCAPG2 as an Immune-Related Prognostic Biomarker in Breast Cancer
Source: World J Oncol. 2026 Jun 25;17(4):509–23. doi: 10.14740/wjon2768 (PMC13375428; doi:10.14740/wjon2768)

Suppl5. The prognosis analyses of the ten hub genes(A-J).


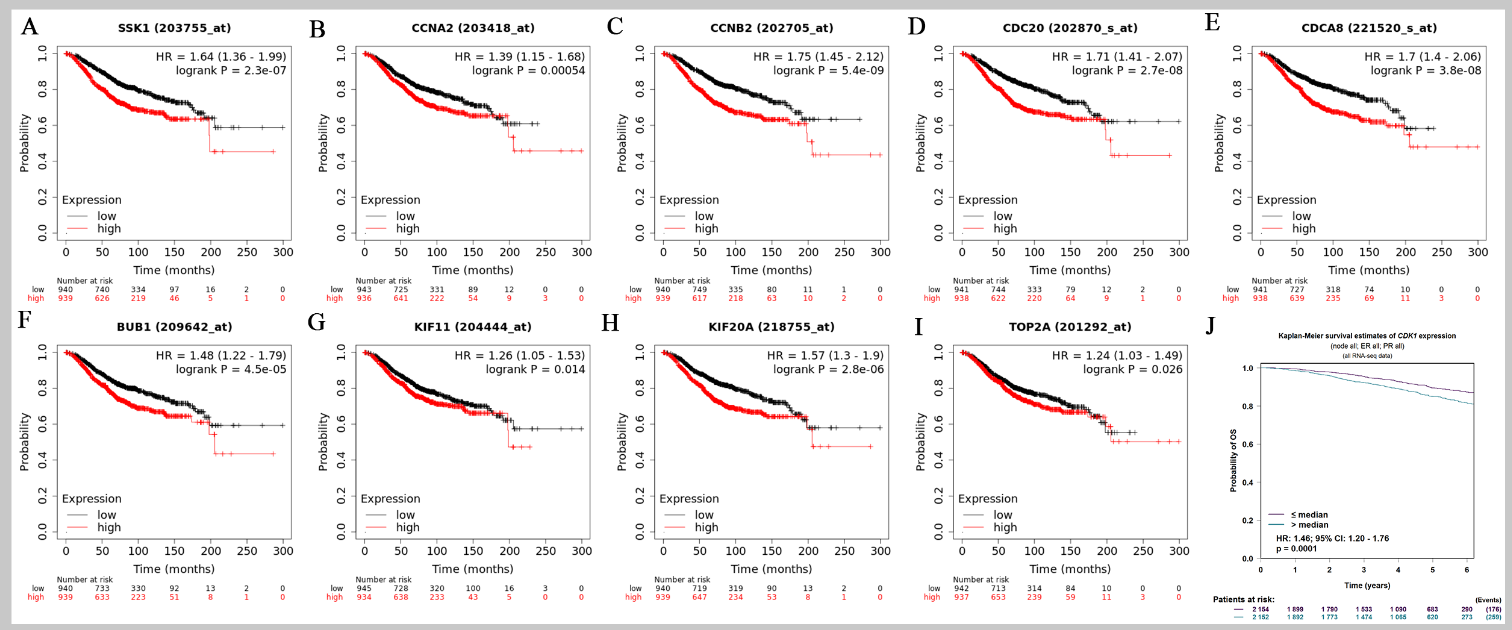

Supplement: Suppl 5 — The prognosis analyses of the 10 hub genes (A–J). [file wjon-17-04-509-s005.docx]
